# Treatment of Multi-Walled Carbon Nanotubes with Dichromic Acid: Oxidation and Appearance of Intercalation

**DOI:** 10.3390/membranes13080729

**Published:** 2023-08-12

**Authors:** Valeriy Golovakhin, Ekaterina Yu. Kim, Oksana N. Novgorodtseva, Evgene A. Maksimovskiy, Arina V. Ukhina, Arcady V. Ishchenko, Alexander G. Bannov

**Affiliations:** 1 Department of Chemistry and Chemical Engineering, Novosibirsk State Technical University, 630073 Novosibirsk, Russia; golovaxin-valera@mail.ru (V.G.); katerina_kim95@mail.ru (E.Y.K.); o.novgorodceva@corp.nstu.ru (O.N.N.); 2 Institute of Solid State Chemistry and Mechanochemistry, Siberian Branch of Russian Academy of Sciences, 630092 Novosibirsk, Russia; auhina181@gmail.com; 3 Institute of Inorganic Chemistry, Siberian Branch of Russian Academy of Sciences, 630090 Novosibirsk, Russia; eugene@niic.nsc.ru; 4 Boreskov Institute of Catalysis, Siberian Branch of Russian Academy of Sciences, 630090 Novosibirsk, Russia; arcady.ishchenko@gmail.com

**Keywords:** carbon nanotubes, functionalization, chemical functionalization, dichromic acid, carbon nanomaterials, MWCNTs, treatment, intercalation, chromium oxide

## Abstract

This work is dedicated to the study of the treatment of multi-walled carbon nanotubes (MWCNTs) with dichromic acid. The dichromic acid was formed by dissolving different concentrations of CrO_3_ in water. The effect of the concentration of dichromic acid on the change in texture characteristics, elemental composition, defectiveness, graphitization degree, and surface chemistry of MWCNTs was investigated using various analytical techniques, such as transmission electron microscopy, energy-dispersive X-ray spectroscopy (EDX), Fourier transform infrared spectroscopy (FTIR), X-ray diffraction, and X-ray photoelectron spectroscopy (XPS). Testing of MWCNTs as electrodes for supercapacitors in 3.5 M H_2_SO_4_ solution was carried out using cyclic voltammetry. A decrease in the average diameter of CNTs after treatment was found. The EDX and XPS showed that the oxygen content on the surface of MWCNTs increased after treatment with dichromic acid. The formation of Cr_2_O_3_ after treatment with dichromic acid was detected by XPS. High angle annular dark field scanning transmission electron microscopy was used to confirm the intercalation of the chromium-containing compound between graphene layers of MWCNTs after treatment with dichromic acid. It was found that two different types of MWCNTs showed diverse behavior after treatment. The highest specific capacitance of the MWCNTs after treatment was 141 F g^−1^ (at 2 mV s^−1^) compared to 0.3 F g^−1^ for the untreated sample.

## 1. Introduction

The study of the effect of oxidants on the change in structure and properties of carbon nanomaterials is an urgent task. More than 300 papers devoted to various methods of carbon nanomaterials functionalization are published annually. Carbon nanomaterials have a wide range of applications in various fields and their research is cutting edge nowadays. The main prospective materials from this group are carbon nanotubes, which are interesting to be applied in supercapacitors [1,2,3,4], membranes [5,6], biosensors [7], polymer composites [8,9], batteries [10], gas sensors [11,12,13], etc. The creation of novel high-performance supercapacitors is on the way to large-scale production and extensive research should be carried out to overcome many problems towards this direction. One of the problems is the low amount of functional groups in as-received carbon nanotubes that hinders their practical use in supercapacitors. The method to modify single-walled carbon nanotubes [14,15] and multi-walled carbon nanotubes (MWCNTs) [16] is chemical treatment, which makes it possible to considerably improve the physical and chemical properties [17,18,19]. There are a variety of other fields where chemical treatment might be useful, e.g., membrane technology [6,20,21], where the selectivity and permeability of CNT-based membranes can be controlled via functionalization.

There are several methods to modify the carbon nanotubes (CNTs), e.g., chemical treatment [22,23], electrochemical treatment [24,25], ball milling [26,27,28], heat treatment [29,30], etc. In [31], the authors considered the prospects for chemical modification of MWNTs as electrodes for cotton textile supercapacitors, indicating the formation of redox-active oxygen groups on the surface of CNTs which induced pseudocapacitive properties. In [32], acid etching was used to open the tips of CNTs and led to the formation of functional groups and enhanced the energy density of the symmetric supercapacitor. At the same time, there are a lot of challenging problems for large-scale chemical treatment of CNTs, and the main one is the loss of material as a result of etching [33]. Unfortunately, there is no systematic research directed to increase the yield of CNTs after chemical treatment. The acid treatment can be considered as the most scalable and convenient method, and for one needs extended data on its technological parameters.

This work was devoted to chemical treatment of MWCNTs with dichromic acid. The latter was obtained by dissolution of CrO_3_ in water; the effect of the concentration of solution was investigated. The strong oxidation of CNTs with dichromic acid, and the decrease in their diameter was detected. The appearance of intercalation in MWCNTs via treatment with dichromic acid was detected. A significant enhancement of specific capacitance of MWCNTs in sulfuric acid electrolyte was found.

## 2. Materials and Methods

### 2.1. Treatment of MWCNTs

Commercial MWCNTs were chemically treated in dichromic acid. Chromic anhydride was added to water to obtain chromic and dichromic acids. Chromate and dichromate ions transfer into each other when the pH of the solution changes. However, in aqueous solutions, when a large amount of CrO_3_ is dissolved in water, it forms mainly dichromic acid (H_2_Cr_2_O_7_; concentration of more than 100 g‧L^−1^ or 0.95 M). We took solutions with concentrations ranging from 1 to 6 mol‧L^−1^. The concentration is indicated in terms of CrO_3_ dissolved in water. The name of the sample was accompanied with the concentration of solution taken for treatment. For example, MWNT-1020_3M sample (Table 1) shows that the sample was treated with 3 mol‧L^−1^ concentration of CrO_3_ dissolved in water.

Commercial multi-walled carbon nanotubes (Shenzhen Nanotech Port Co., Shenzhen, China) were investigated. There are two samples marked as MWNT-1020 and MWNT-4060, respectively.

The treatment was carried out as follows. A sample of pristine MWCNTs (0.15 g) was placed in a conical flat-bottomed flask, and then 100 mL of CrO_3_ solution was poured and heated for 6 h with constant stirring at 80 °C. After 6 h of boiling, the treated sample was washed with distilled water until pH = 7 with different indicators (Cresol red [II], Bromothymol blue, Methyl Red, test paper) and vacuum filtered. After filtration, the treated MWCNTs were dried at 100 °C for 12 h. After drying, the samples were ground in a mortar and sifted through a 100-µm mesh size sieve.

### 2.2. Characterization

Transmission electron microscopy (TEM) was carried out using a JEM-2200FS CS (JEOL, Tokyo, Japan) microscope. The elemental composition of carbon materials after treatment was investigated using a scanning electron microscope S–3400N (Hitachi, Tokyo, Japan) equipped with an add-on for energy dispersive X-ray spectroscopy (EDX) manufactured by Oxford Instruments Co. The samples were investigated without sputtering (electron beam energy 10 keV), with a Li-Si detector at an elevation of detector 35° and a 0° inclination of the sample.

The defectiveness of carbon nanomaterials was estimated using Raman spectroscopy on the device T64000 «Horiba Jobin Yvon» (Ar laser, λ = 514 nm). The disorder degree of carbon nanomaterials was estimated from the ratio of intensities of D and G peaks [34]. In addition to Raman spectroscopy, the structural features of carbon nanomaterials were also determined by means of X-ray diffraction (XRD) using DRON-3 diffractometer (Russia) and Cu Kα radiation (λ = 1.54 Å). The degree of graphitization Y of carbon nanomaterials was calculated on the basis of interlayer spacing d_002_, which, in turn, corresponds to the main plane of graphite [35] according to Equation (1) shown below.

Y = (3.440 − d_002_)/(3.440 − 3.354), (1)

where 3.440 Å is an interlayer spacing of turbostratic structure carbon, and 3.354 Å is an interlayer spacing in graphite (defect-free material).

Fourier transform infrared spectroscopy (FTIR) was used for analysis of functional groups in MWCNTs and carried out using FT-801 spectrometer (Simex, Novosibirsk, Russia).

The analysis of functional groups formed as a result of treatment was also studied using X-ray photoelectron spectroscopy (XPS). The chemical composition of the surface of the samples was studied using a SPECS Surface Nano Analysis GmbH (Berlin, Germany) spectrometer. The spectrometer was equipped with hemispherical analyzer PHOIBOS-150 and an XR-50 X-ray characteristic radiation source with a double Al/Mg anode. Non-monochromatic Al *K*_α_ radiation (h* = 1486.61 eV) was used. Relative concentrations of elements within the area of analysis were determined based on the integral intensities of XPS lines taking into account the cross section of photoionization of corresponding terms. For detailed analysis, we used decomposition of spectra into individual components. Accordingly, after background subtraction by the Shirley method, the experimental curve was decomposed into a set of lines corresponding to the photoemission of electrons from atoms in a different chemical surrounding. The data were processed using the CasaXPS software (http://www.casaxps.com/). The shape of peaks was approximated by a symmetric function obtained by multiplication of the Gaussian and Lorentzian functions. C1 s peak (284.50 eV) corresponding to carbon in sp^2^ hybridization (graphite, graphene) was used to account for the charging effect.

High angle annular dark field scanning transmission electron microscopy (HAADF-STEM) micrographs were obtained with the Themis-Z 3.1 instrument (TFS, Plano, TX, USA) equipped with an X-FEG-monochromator and CS/S double corrector. Accelerating voltage was 200 kV. Elemental analysis was performed with a Super-X EDS detector (energy resolution about 120 eV). The samples for the study were prepared by ultrasonic dispersing in ethanol and consequent deposition of the suspension upon a “holey” carbon film supported on a copper grid.

### 2.3. Supercapacitors

The chemically treated MWCNTs were tested as electrodes for supercapacitors. To calculate the specific capacitance of the studied samples, the cyclic voltammetry at different rates of sweep rates (2–10 mV∙s^−1^) was carried out. Voltammetry curves were recorded using IPC-compact potentiostat (Russia). Three-electrode scheme was used for measurements: auxiliary electrode (Pt), reference electrode (Ag/AgCl; saturated KCl), working electrode made using MWCNTs. Potentials are given relative to the Ag/AgCl electrode. The specific capacitance of the carbon materials under study was determined using the Formula (2) [36,37,38,39,40,41]:

(2) $$C_{sp}=\frac{J}{Vm}$$

where C_sp_ is the specific capacitance, F‧g^−1^; J is the sum of cathodic and anodic currents (J = J_k_ + J_a_) at 500 mV, mA; m is the weight of carbon material, g; V is a sweep rate, mV‧s^−1^. MWNT-1020 and MWNT-4060 samples were mixed with 10–15% carbon black (Alfa Aesar, Haverhill, MA, USA), 0.01 g of the resulting composite was taken and mixed with 10% vaseline oil (Russia) to form a pasty state. The resulting mixture was evenly applied to the graphite electrode (S = 1 cm^2^). Then, the electrodes were immersed in 3.5 M H_2_SO_4_ solution and cyclic voltammetry curves were recorded using direct voltammetry when the electrical potential at the working electrode changed from 0 to 1 V. The error of the sweep rate was 1.0%; the error of the potential setter was 0.03 mV.

## 3. Results and Discussion

**Figure 1:**
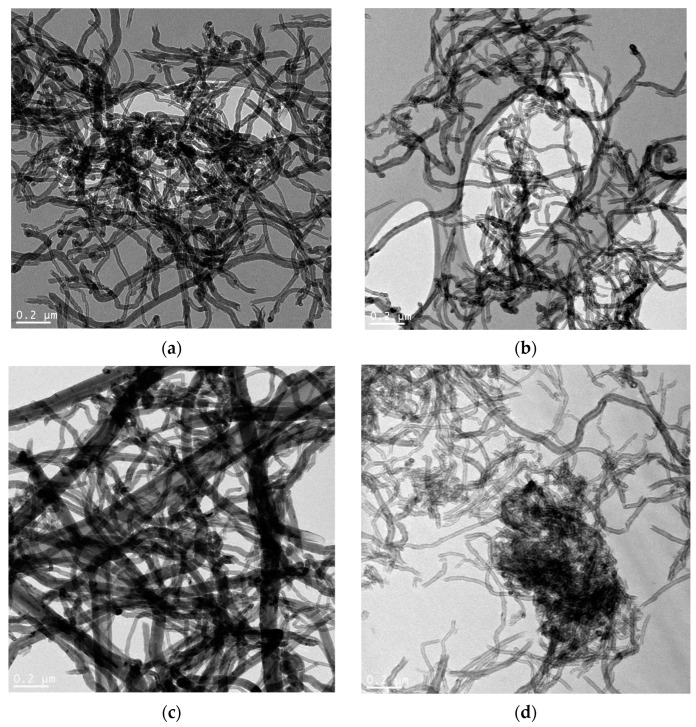

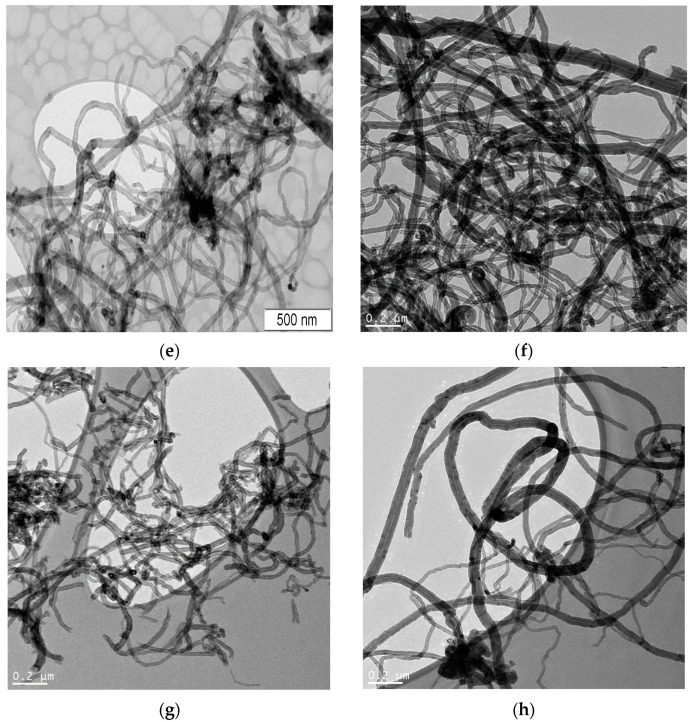
TEM images of treated MWCNTs: (**a**) non-treated MWNT-1020; (**b**) MWNT-1020_1M; (**c**) MWNT-1020_3M; (**d**) MWNT-1020_6M; (**e**) non-treated MWNT-4060; (**f**) MWNT-4060_1M; (**g**) MWNT-4060_3M; (**h**) MWNT-4060_6M.

TEM images of chemically modified MWCNTs are shown in Figure 1. Images of pristine MWCNTs were presented in [42] (MWNT-1020 sample) and [43] (MWNT-4060 sample). The difference in the number of layers in CNTs of two samples can be seen. The chemical treatment of MWCNTs induced the shortening of carbon nanotubes, which is a typical effect in chemical treatment of carbon nanomaterials [16]. Additional high-resolution TEM images of treated MWCNTs are presented in Supplementary Materials (Figure S1). The catalytic particles partially remained in both samples within the channel inside the CNTs (Figure S1b in Supplementary Materials). At the same time, there are a lot of empty cups in nanotubes observed in chemically treated materials (probably, the treatment was carried out by manufacturer).

Based on the TEM micrographs of the samples, the diameters of MWCNTs (Table 2) were calculated. It was found that the average diameter of carbon nanotubes decreased after chemical treatment. This can be induced by etching of graphene layers on the surface of carbon nanotubes. The increase of concentration of dichromic acid led to a decrease in average diameter of the CNTs. It is worth noting that the etching of surface graphene layers was stronger for MWNT-1020 compared to MWNT-4060 samples. Finally, the average diameter of carbon nanotubes treated with 6M H_2_Cr_2_O_7_ was close to each other. The decrease in diameter of CNTs was also reported in [44] for sulfuric acid treatment.

The distribution of diameter of CNTs formed during the treatment is shown in Figure S2 in Supplementary Materials. From the distribution curves of MWNT-1020 samples, it is seen that the increase in concentration of dichromic acid led to a shift of the maximum of the distribution curve from the 25–30 nm range to 20–25 nm and even 15–20 nm. The same behavior was observed for the MWNT-4020 sample, showing a shift of the maximum of distribution to the 10–20 nm range. Concerning the larger diameters, it is seen that the maximum diameters of MWNT-1020 and MWNT-4060 were 70 nm and 120 nm, respectively. The treatment showed a decrease of the span of distribution to maximum values of 50 nm and 100 nm.

**Figure 2:**
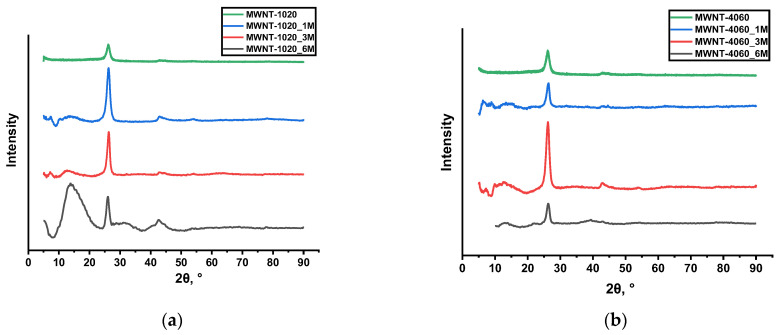
XRD spectra of the MWCNTs (Cu Kα radiation) treated with dichromic acid: (**a**) MWNT-1020; (**b**) MWNT-4060.

Figure 2 shows X-ray diffraction patterns of the treated samples. XRD patterns of non-treated MWCNTs were reported in [43]. The presence of (002) reflection typical for CNFs around 2θ~26° was shown. At the same time, there are reflections at the angle 2θ = 10–15°. Strong reflections were especially observed for the MWNT-4060_1M sample. These peaks were also for all samples treated with H_2_Cr_2_O_7_. The reflection around 2θ = 10–15° was relatively weak at the concentration of 3M (2θ~13° for both types of MWCNTs) and the MWNT-4060_6M sample (2θ = 12.8° and 14.7° for MWNT-1020 and MWNT-4060 samples, respectively). The appearance of such reflections with interlayer spacing higher than d_002_ can be considered as the formation of intercalation compounds, since such an effect usually causes an increase of interlayer spacing [45,46]. In CNTs, it is clearly seen for intercalation with chlorides [47,48] and other compounds [49,50]. It is worth noting that there were no reflections related to chromium oxides or any other compounds in the XRD patterns.

**Figure 3:**
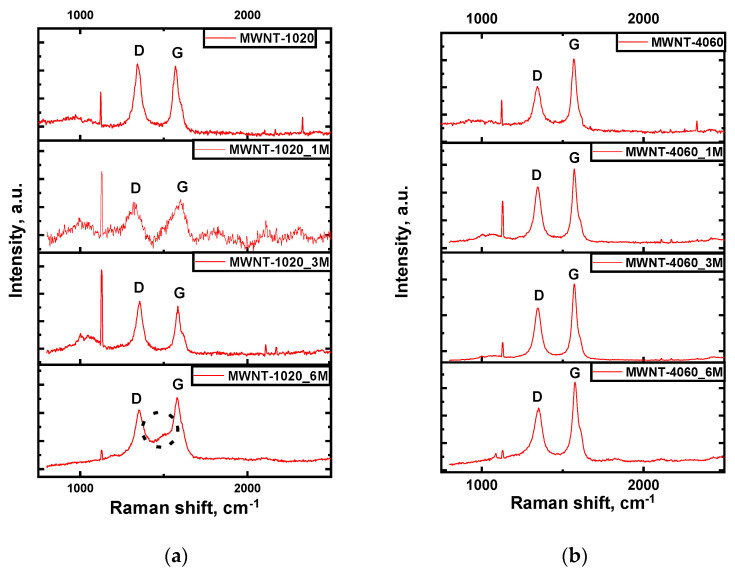
Raman spectra (λ = 514 nm) of the MWCNTs: (**a**) MWNT-1020; (**b**) MWNT-4060.

Raman spectroscopy provides information related to structural changes in nanotubes and can be a direct evidence of chemical functionalization [27,51]. Raman spectra obtained for the non-treated and functionalized MWCNTs are shown in Figure 3.

Two main bands were observed; the D band, which results from the formation of sp^3^-bonded carbon atoms, at approximately 1340 cm^−1^ and the G band, which is related to the tangential modes of the sp^2^-bonded carbon atoms, at approximately 1570 cm^−1^ [34,52]. The D band is a two-resonance Raman mode, which is influenced by defects in the graphene structure. This band together with the G-band can be used for material characterization to probe and monitor the structural modifications of the nanotube sidewalls resulting from the introduction of defects and the attachment of various molecules. All functionalized materials showed an increase in the I(D)/I(G) ratio compared to the non-modified samples (Table 3). The only exclusion is the MWNT-1020_6M sample, which was strongly oxidized and showed the highest concentration of chromium according to EDX. The direct proof of stronger oxidation of samples in 6M H_2_Cr_2_O_7_ solutions is the lowest average diameter of CNTs (according to TEM) and graphitization degree Y (according to XRD). Usually, the increase of I(D)/I(G) after acid treatment takes place [44,53] and the cases of its decrease compared to pristine material are rare, but in such papers the initial defectiveness of MWCNTs is low enough (for example, I(D)/I(G) = 0.2 [53] or I(D)/I(G) = 0.47 [44]) compared to this paper. The surface area of both types of MWCNTs tends to decrease when exposing dichromium acid solution treatment (Table 3). Usually, the surface area of CNTs grows during chemical treatment [54]; however, it can be assumed that the surface layers of nanotubes were actively etched with dichromic acid leading to removal of defective surface layers. This effect apparently induces the slight change in specific surface area.

The increase of I(D)/I(G) indicates the growth of the disorder degree of material caused by chemical treatment. The interesting behavior was found in the MWNT-1020_6M sample, showing the appearance of a new additional peak in the Raman spectrum between D and G modes (1487 cm^−1^). Such a peak between two main modes is usually seen in spectra of graphite intercalation compounds [55]. The formation of intercalation compounds is also confirmed by the additional peak at 2θ = 10–15° in XRD patterns. Such a peak is observed in all chemically treated samples.

**Figure 4:**
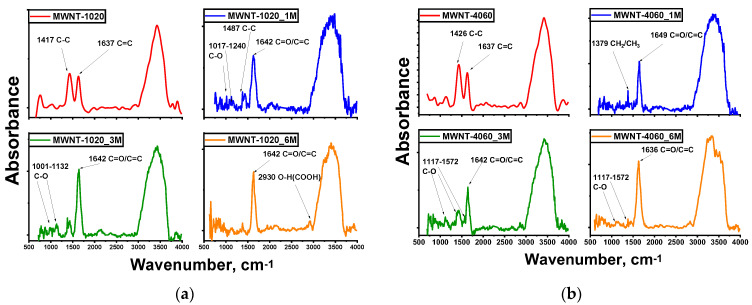
FTIR spectra of MWNTs: (**a**) MWNT-1020; (**b**) MWNT-4060.

FTIR spectra are shown in Figure 4. Also for this treatment, a peak on the 2930 cm^−1^ absorption band corresponding to the valence vibration of the O-H group in carboxyls is observed [56]. For less concentrated solutions in carbon materials, C-O (carboxyl, ethers) and C=O (carboxyl) valence vibrations are observed in the 1017, 1132, 1240 cm^−1^ absorption bands [16,36,57]. This is due to the fact that less concentrated solutions do not etch the MWCNTs’ surface and allow more diverse groups to form. For MWNT-4060 C-C and C=C, stretching vibrations are observed; as for MWNT-1020 samples, however, stretching vibrations of C-H bonds were still recorded. In this case, CNTs treated with 3M solutions were better functionalized, since the most diverse valence vibrations were detected.

**Figure 5:**
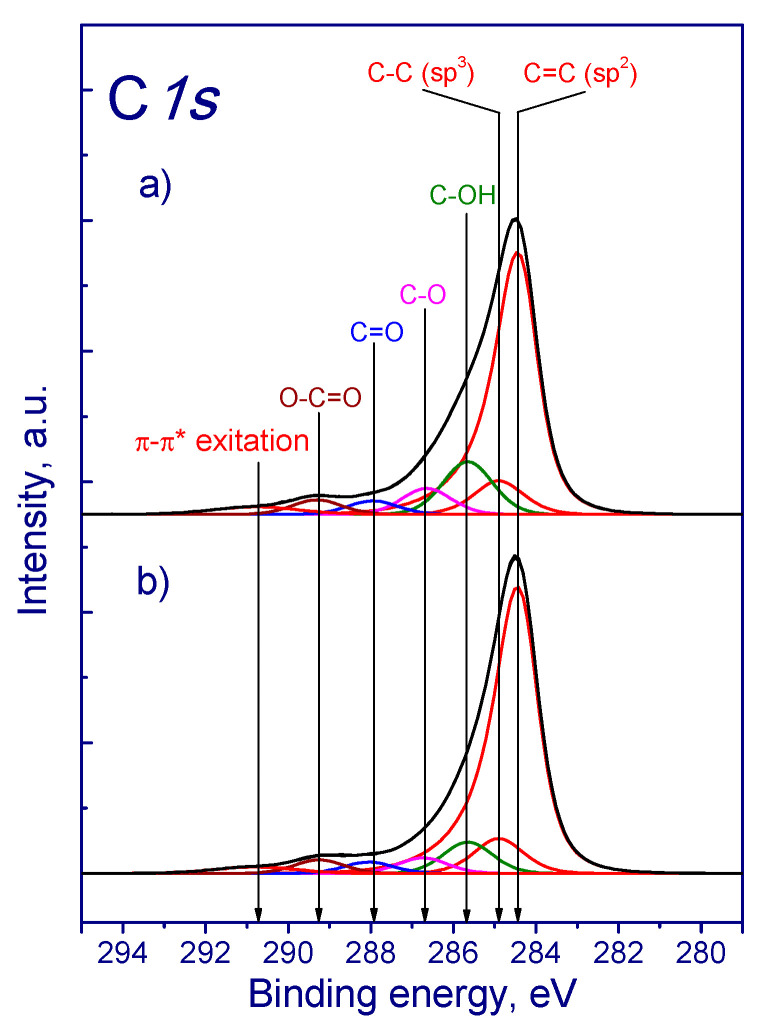
C1s spectra of samples: (**a**) MWNT-4060_6M, (**b**) MWNT-1020_6M.

XPS showed the significant oxidation of samples as a result of treatment with dichromic acid. Figure 5 shows the typical C1s spectra of the samples studied.

**Figure 6:**
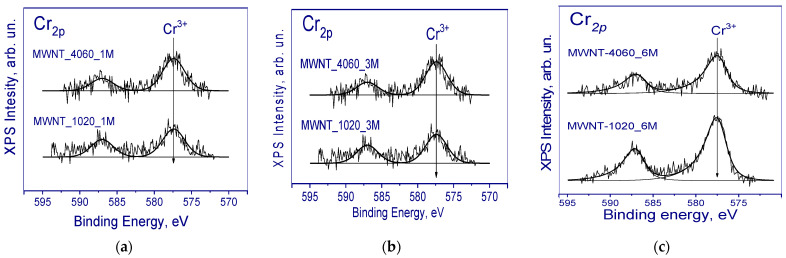
Cr_2p_ photoelectron spectra of MWCNTs treated with different concentrations: (**a**) 1M; (**b**) 3M; (**c**) 6M CrO_3_ solution.

C1s spectra can be described by several peaks corresponding to carbon in different chemical surroundings. Thus, the peak around 284.5 eV corresponding to carbon in graphite (sp^2^ hybridization) was used as an internal standard to account for the charging effect of the samples. In addition, the spectrum of carbon nanotubes contains peaks around 289.7 and 290.9 eV. According to the literature data, the peaks in the region of 286.4-284.8, 288.1, and 288.8 eV can be attributed to carbon bonded to oxygen, e.g., C-O, C=O, and O-C=O, respectively [58,59,60,61]. Since the treatment of MWCNTs was carried out in the chromium-containing compound, the presence of chromium was detected. The Cr_2p_ spectra are shown in Figure 6.

In the Cr_2p_ spectrum of the studied samples, the Cr2p_3/2_-Cr2p_1/2_ doublet was observed. The binding energy of Cr2p_3/2_ was 577.6 eV, which is typical for chromium in the Cr^3+^ state. In the literature, the Cr2p_3/2_ binding energy value of 576.6–577.0 eV is given for bulk Cr_2_O_3_ oxide [62]. For Cr_2_O_3_ oxide deposited on Al_2_O_3_, the binding energy of Cr2p_3/2_ was 577.2–577.6 eV. CrO_3_ oxide is characterized by a significantly higher binding energy of Cr2p_3/2_ in the range of 579.1–580.0 eV [63]. Therefore, it is assumed that during the interaction of the dichromic acid with carbon nanotubes, carbon played the role of a reducing agent converting H_2_Cr_2_O_7_ to Cr_2_O_3_, which most likely occurred somewhere inside the structure after the formation of intercalation compounds (GICs) took place. Other C1*s* spectra are shown in Figure S4 (Supplementary Materials). It is worth noting that there is no formation of chromium oxide nanoparticles seen in the TEM images (Figure S1 in Supplementary Materials and Figure 1). The formation of graphite intercalation compounds with chromium oxide was already reported, but only in a few papers. In [64], the formation of CrO_3_-GICs (i.e., graphite intercalation compounds) was discussed during treatment of graphite in CrO_3_ and 12 M HCl solution. The authors suggested not only doping with CrO_3_ after its interaction with SWCNTs, but also its intercalation between graphene layers [64].

The proposed reaction of dichromic acid reduction to chromium (III) oxide is shown below:

3C + 2H_2_Cr_2_O_7_ = 2Cr_2_O_3_ + 3CO_2_ + 2H_2_O

**Figure 7:**
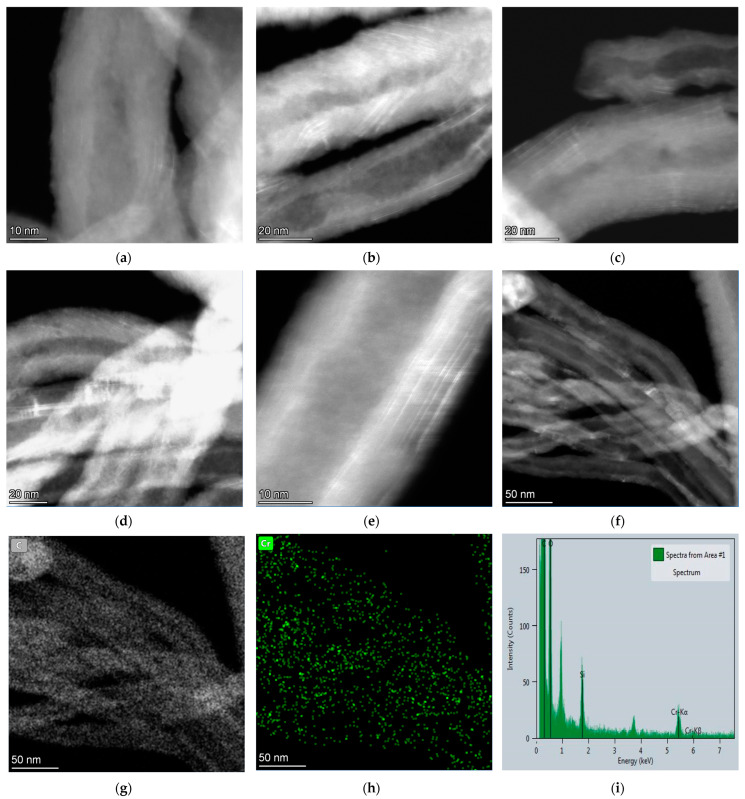
(**a**–**f**) HAADF-STEM micrographs of MWNT-1020_6M; (**g**,**h**) EDX mapping of image (**f**) and (**i**) EDX spectrum obtained from area of image.

According to XPS, the presence of chromium in the Cr^3+^ state, the additional peak in Raman spectra, and the reflections within a range 2θ = 10–20° in XRD patterns confirm that there is a formation of Cr_2_O_3_ intercalated compound between graphene layers of MWCNTs chemically treated with dichromic acid. These data were also confirmed by HAADF-STEM images of MWNT-1020_6M sample depicted in Figure 7. The presence of chromium in the sample is shown by the bright traces in the micrographs, since the mass of carbon atoms is significantly lower compared to chromium. The typical lines of chromium distribution of the sample along the graphene layers are clearly seen (Figure 7b,c,e). EDX mapping of the Figure 7i image showed the presence of C, O, Si, and Cr elements. The appearance of silicon in the EDX spectrum is related to mesh substrate.

**Figure 8:**
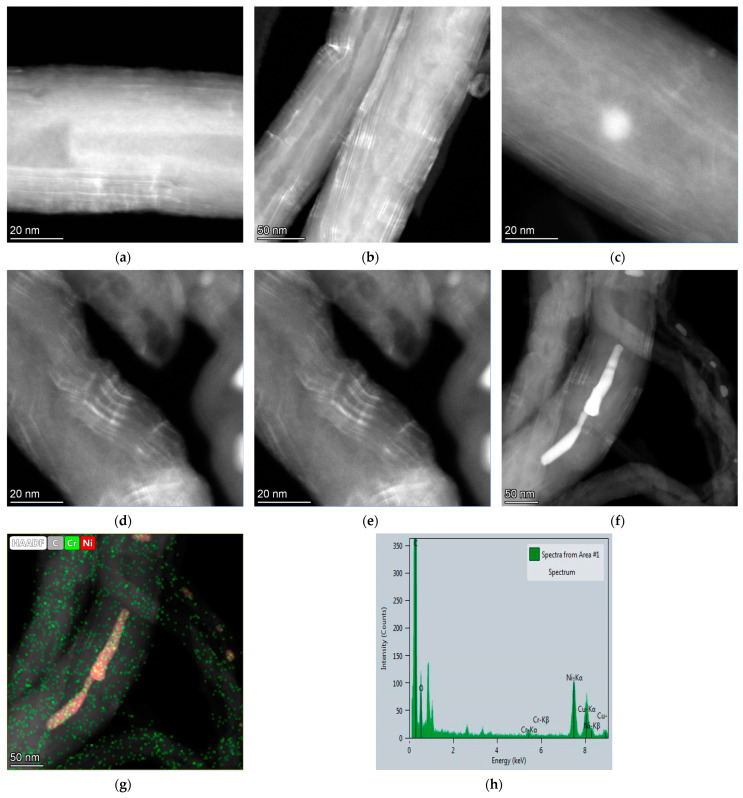
(**a**–**f**) HAADF-STEM micrographs of MWNT-4060_3M; (**g**) EDX mapping and (**h**) EDX spectrum obtained from area of image (**f**).

Moreover, in order to clearly confirm the presence of chromium in the form of the Cr_2_O_3_ intercalated compound, the HAADF-STEM images of other group of samples, e.g., MWNT-4060_3M were taken (Figure 8). It is shown that even there are a weak peak within 2θ = 10–20° in XRD patterns of samples treated with dichromic acid of both types of MWCNTs investigated in this paper, the intercalation of chromium-containing compounds takes place. The detection of chromium along with nickel coming from catalytic nanoparticles in the CNTs has been shown. Additional HAADF-STEM micrographs and EDX spectra are presented in Supplementary Materials (Figure S3).

One considering the content of other elements, Table 4 shows the binding energies of the C1s photoelectron peaks and their concentration in spectra. The highest concentration of the C-OH (285.7 eV) component of C1s spectrum was detected. The content of components related to carbon bonded to oxygen (C-OH, C-O, C=O, O-C=O) decreased predominantly when increasing the concentration of dichromic acid. There is some exclusion as MWNT-4060_3M showed the highest concentration of C-OH groups (Table 4).

Data on content of elements in the sample are presented in Table 5. According to EDX, the C:O ratio decreases when increasing the concentration of solutions. This was shown for both MWCNT samples. EDX showed a higher C:O ratio, whereas XPS showed growth of this value for the MWNT-4060 sample after treatment. The surface nature of analysis (for XPS) showed the higher oxidation degree of the surface compared to EDX. The presence of chromium was detected in all samples treated. It is worth noting that the non-treated sample did not contain the chromium. The highest concentration of chromium is also in agreement with the appearance of indicators of intercalation by Raman spectroscopy and XRD.

The behavior of chemically treated MWCNTs as electrodes of supercapacitors was carried out. Cyclic voltammetry at low sweep rates makes it possible to estimate the capacitance of the material as a whole, not just the contribution of the surface layer. Table 6 shows the values of specific capacitance for the materials under study.

The difference in capacitance for the two types of materials, despite the high degree of functionalization and chromium content in all samples, is more related to the structure of the material. The use of dichromic acid treatment leads not only to etching, but also to the formation of a large number of functional groups. It is observed that the chromium content increases when increasing the solution concentration, but this does not explain the difference in the increase in specific capacitance for the 1M solution compared to the 3M one. This difference is more due to the difference in the content of functional groups formed since the specific surface area did not change significantly during the treatment (Table 3). Pore size distribution curves of the samples with the highest capacitance are presented in Figure S5 in Supplementary Materials. The low concentration of dichromic acid does not lead to etching of CNTs surface and leaves more functional groups. The increase in concentration to 3M led to strong etching and delamination of surface graphene layers, which was confirmed by the graphitization degree for MWCNTs, which became higher (e.g., 67% for MWNT-1020_3M sample) compared to untreated ones (46.5% for MWNT-1020 sample). In addition, the samples treated in the 3M H₂Cr₂O₇ solution showed the highest disorder degree according to Raman spectroscopy.

When comparing the specific capacitance of MWCNTs treated with sulfuric acid, which is one of the most frequently used oxidizers [44], it can be concluded that the treatment with dichromic acid leads to higher specific capacitance of material as well as lower C:O ratios [2]. Moreover, sometimes the oxidative treatment with sulfuric acid or nitric acid does not lead to a significant improvement in specific capacitance (the latter varied from 2.9 to 3.67 F g^−1^ for chemically treated CNT-based textile samples) [31].

**Figure 9:**
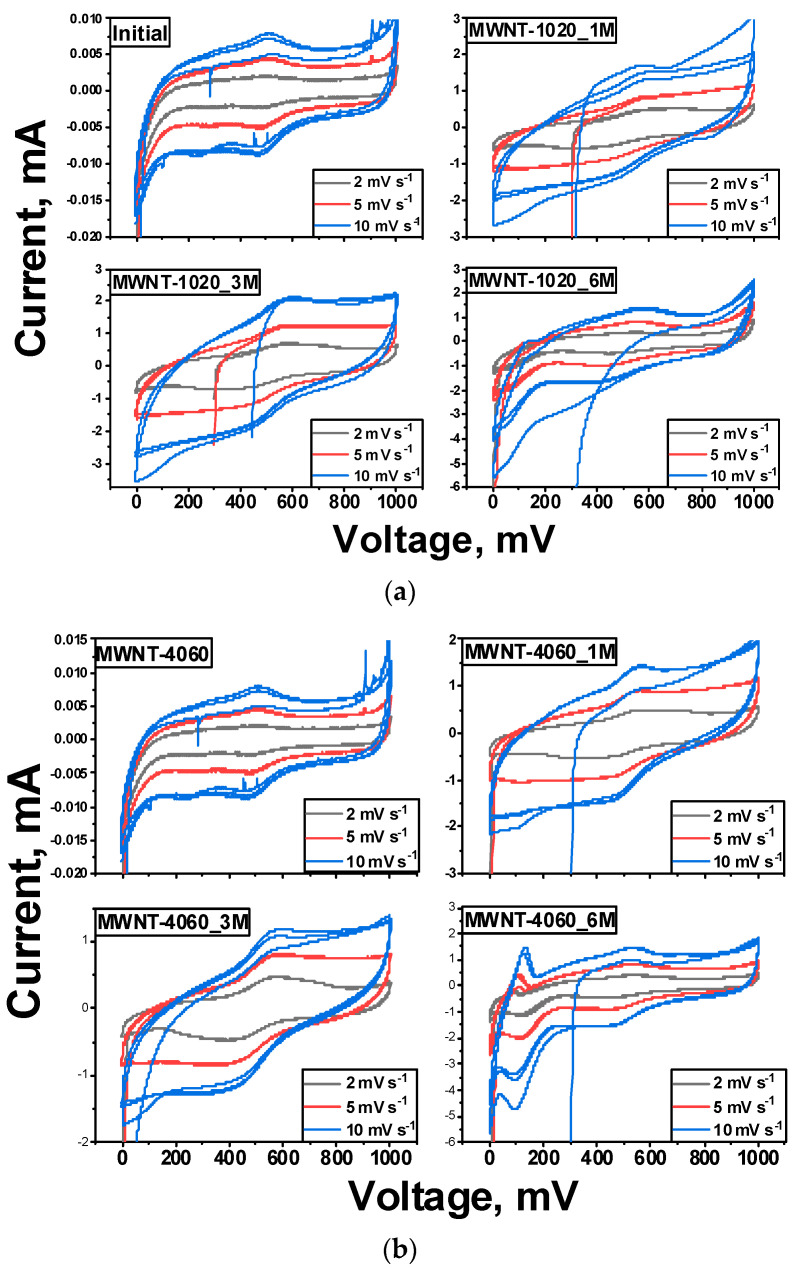
Cyclic voltammetry curves of MWCNTs treated with dichromic acid: (**a**) MWNT-1020; (**b**) MWNT-4060.

Figure 9 shows cyclic voltammetry curves showing peaks at 480–530 mV, corresponding to redox processes occurring with oxygen in functional groups (namely the oxidation and reduction of the hydroxyl and carbonyl group –CHO ↔ COH at a potential ~500 mV) [65]. The peaks around 500 mV were recorded for the entire set of treated samples. It is worth noting that the current spikes at 100 mV for the MWNT-4060_6M sample were observed, but there are no reported data describing processes at this voltage.

The specific capacitance taken at low sweep speeds reflects to a greater extent the capacitance of the material itself, however, we cannot ignore the rich group composition on the surface layer. For MWNT-1020, lower solution concentrations result in the highest number of functional groups. Even at low total oxidation degree in the sample treated in a 6M solution, there is a large amount of chromium (1.6 at.%), which, in turn, influenced a sharp jump in capacitance, associated precisely with the intercalation of Cr_2_O_3_. The MWNT-4060 sample possessed a lower disorder degree compared to MWNT-1020, and the first is oxidized but not as strong. It is worth noting that for the 6M solution, there is over-etching of the surface, leading to both peeling of graphene layers from the surface (structural damage), and the oxidation of the already existing groups to CO or CO_2_ predominantly. Because of this, the specific capacitance of MWNT-4060_6M was very low compared to other treated samples. The impact of surface area on the enhancement of the capacitance of MWCNTs is negligible, since the treatment with dichromic acid did not lead to an increase in this value (Table 3).

Overall, the study demonstrated that the treatment of MWCNTs with dichromic acid using various concentrations can significantly improve their texture characteristics, elemental composition, and surface chemistry. This treatment can be useful for various applications of MWCNTs, such as in membranes, energy storage, catalysis, gas sensors, and biomedical applications.

## 4. Conclusions

Two different brands of MWCNTs were used for investigation, and they showed different behavior in dichromic acid solutions. The decrease in average diameter of the treated carbon nanotubes was found. The main result of this paper is the strong oxidation of CNTs and intercalation of chromium (III) oxide into multi-walled carbon nanotubes after treatment with dichromic acid that was revealed by XRD, Raman spectroscopy, and XPS. The concentration of chromium in the sample was at the level of 0.1–1.68 at.% (according to EDX). At the same time, TEM images did not show the formation of any other inclusions in the samples except for MWCNTs. The treatment with dichromic acid made it possible to significantly improve the specific capacitance of MWCNTs in H_2_SO_4_ electrolyte and reach the values above 70 F/g (at 10 mV s^−1^). The study demonstrated that the treatment of MWCNTs with dichromic acid using various concentrations can significantly improve their physical and chemical properties. The treatment can be useful for various applications of MWCNTs in membranes, energy storage, catalysis, gas sensors, and biomedical applications.

## Figures and Tables

**Table 1:** Samples and preparation technique.

Sample	Concentration of CrO_3,_ mol‧L^−1^
MWNT-1020	-
MWNT-1020_1M	1
MWNT-1020_3M	3
MWNT-1020_6M	6
MWNT-4060	-
MWNT-4060_1M	1
MWNT-4060_3M	3
MWNT-4060_6M	6

**Table 2:** Average diameter of MWCNTs.

Sample	Average Diameter of CNTs, nm
MWNT-1020	26 ± 0.82
MWNT-1020_1M	25.6 ± 0.75
MWNT-1020_3M	23.9 ± 1.42
MWNT-1020_6M	21.4 ± 0.55
MWNT-4060	36 ± 1.51
MWNT-4060_1M	27.1 ± 1.27
MWNT-4060_3M	23.2 ± 1.82
MWNT-4060_6M	22.6 ± 1.20

**Table 3:** Raman spectroscopy and XRD data.

Sample	Raman Spectroscopy			XRD		Surface Area (BET), m^2^/g
	FWHM, cm^−1^ (D)	FWHM, cm^−1^ (G)	I(D)/I(G)	d_002_, nm	Y, %	
MWNT-1020	52.4	50.6	1.03	0.3400	46.5	128
MWNT-1020_1M	89	78	1.28	0.3380	43.5	123
MWNT-1020_3M	53.6	53.9	1.16	0.3382	67	119
MWNT-1020_6M	61.8	73	0.78	0.3414	30	111
MWNT-4060	50.7	42.5	0.54	0.3387	61.6	68
MWNT-4060_1M	52	44.4	0.70	0.3385	62.3	65
MWNT-4060_3M	54.6	44.4	0.78	0.3367	84	63
MWNT-4060_6M	59	51.7	0.65	0.3388	61	61

**Table 4:** XPS data of MWCNTs chemically treated with dichromic acid.

Sample	Concentration of Components of C1s Photoelectron Peak (at.%)						
	C=C (sp^2^) 284.5 eV	C-C (sp^3^) 284.9 eV	C-OH 285.7 eV	C-O 286.7 eV	C=O 288.0 eV	O-C=O 289.3 eV	π-π* 290.8 eV
MWNT-1020	65.4	8.0	13.2	6.0	2.2	2.8	2.4
MWNT-1020_1M	62.1	8.1	17.4	4.4	2.5	4.2	1.4
MWNT-1020_3M	60.5	16.0	9.0	4.7	3.1	3.3	3.5
MWNT-1020_6M	72.4	8.3	7.3	3.6	2.6	3.2	2.6
MWNT-4060	57.0	4.2	23.0	7.9	2.8	3.9	1.2
MWNT-4060_1M	59.9	11.8	14.8	5.2	2.5	3.7	2.1
MWNT-4060_3M	63.7	6.1	17.1	4.4	2.8	3.8	2.0
MWNT-4060_6M	63.4	9.9	12.3	5.0	3.0	3.4	3.1

**Table 5:** EDX and XPS results of the concentration of elements in the samples.

Sample	EDX		XPS	
	C:O	Impurities, at.%	C:O	Impurities, at.%
MWNT-1020	-	0	13.1	0
MWNT-1020_1M	22	Cr (0.1)	7.3	Cr (0.16)
MWNT-1020_3M	14	Cr (0.31)	7.7	Cr (0.29)
MWNT-1020_6M	10	Cr (1.68)	8.54	Cr (0.19)
MWNT-4060	-	0	9.4	0
MWNT-4060_1M	73	Ni (0.13) Cr (0.12)	8.5	Cr (0.16)
MWNT-4060_3M	33	Ni (0.11) Cr (0.15)	8.53	Cr (0.35)
MWNT-4060_6M	18	Cr (0.48)	6.4	Cr (0.11)

**Table 6:** Cyclic voltammetry results.

Sample	Specific Capacitance C_sp_, F g^−1^		
	10 mV s^−1^	5 mV s^−1^	2 mV s^−1^
MWNT-1020	0.3	0.5	0.7
MWNT-1020_1M	38	42	60
MWNT-1020_3M	35	43	58
MWNT-1020_6M	76	89	111
MWNT-4060	0.16	0.19	0.3
MWNT-4060_1M	66	86	114
MWNT-4060_3M	71	97	141
MWNT-4060_6M	28	33	40

## Data Availability

Not applicable.

## Supplementary Materials

The following supporting information can be downloaded at: https://www.mdpi.com/article/10.3390/membranes13080729/s1. Figure S1. TEM images of treated samples of MWNTs: (a) MWNT-1020_1M; (c) MWNT-1020_3M; (e) MWNT-1020_6M; (b) MWNT-4060_1M; (d) MWNT-4060_3M; (f) MWNT-4060_6M. Figure S2. Distribution of diameters of CNFs formed during the treatment: (a) MWNT-1020 samples; (b) MWNT-4060 samples. Figure S3. HAADF-STEM micrographs of MWNT-4060_3M sample. Figure S4. C1s X-ray photoelectron spectra of treated samples: (a) MWNT-4060_1M; (b) MWNT-1020_1M; (c) MWNT-4060_3M; (d) MWNT-1020_3M. Figure S5. Pore size distribution of MWCNT samples with high specific capacitance: (a) WNT-1020; (d) MWNT-4060 initial samples; (c) MWNT-1020_6M; (d) MWNT-4060_3M chemically treated samples.
