# Supplementary material for: Treatment of Multi-Walled Carbon Nanotubes with Dichromic Acid: Oxidation and Appearance of Intercalation
Source: Membranes (Basel). 2023 Aug 12;13(8):729. doi: 10.3390/membranes13080729 (PMC10456443; doi:10.3390/membranes13080729)
Supplement: Supplementary file 1 [file membranes-13-00729-s001.zip › membranes-2454073-supplementary.pdf]

Supplementary Materials

Treatment of Multi-Walled Carbon Nanotubes with Dichromic Acid: Oxidation and Appearance of Intercalation

Valeriy Golovakhin [1], Ekaterina Yu. Kim [1], Oksana N. Novgorodtseva [1,2], Evgenii A. Maksimovskii [3], Arina Ukhina [2], Arcady V. Ishchenko [4], and Alexander G. Bannov [1,*]

[1] Department of Chemistry and Chemical Engineering, Novosibirsk State Technical University, 630073 Novosibirsk, Russia; golovaxin-valera@mail.ru (V.G.); katerina_kim95@mail.ru (E.Y.K.); o.novgorodceva@corp.nstu.ru (O.N.N.)
[2] Institute of Solid State Chemistry and Mechanochemistry, Siberian Branch of Russian Academy of Sciences, 630092 Novosibirsk, Russia; auhina181@gmail.com
[3] Institute of Inorganic Chemistry, Siberian Branch, Russian Academy of Sciences, 630090 Novosibirsk, Russia; eugene@niic.nsc.ru
[4] Boreskov Institute of Catalysis, Siberian Branch of Russian Academy of Sciences, 630090 Novosibirsk, Russia; arcady.ishchenko@gmail.com
* Correspondence: bannov.alexander@gmail.com

TEM images of treated MWCNTs are presented below.

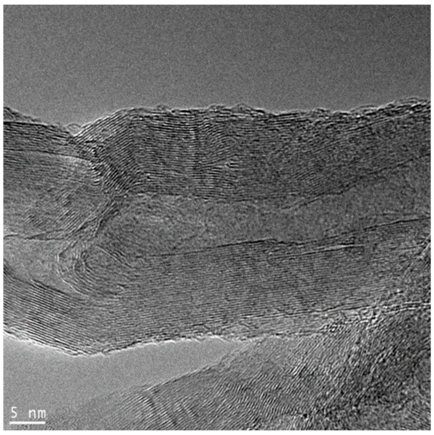

(**a**)

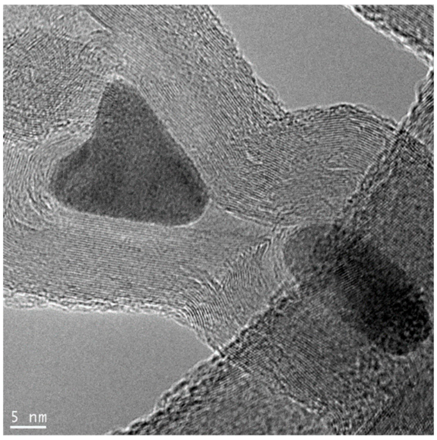

(**b**)

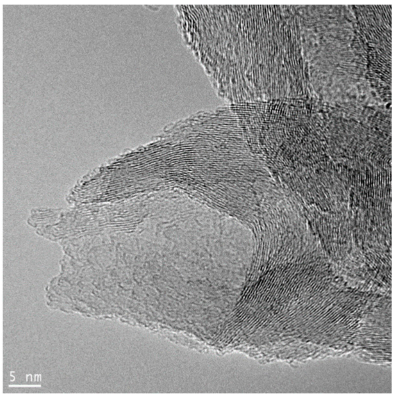

(**c**)

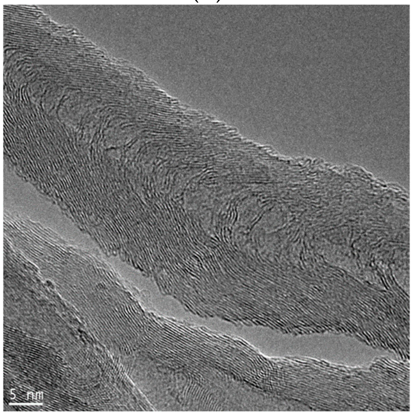

(**d**)

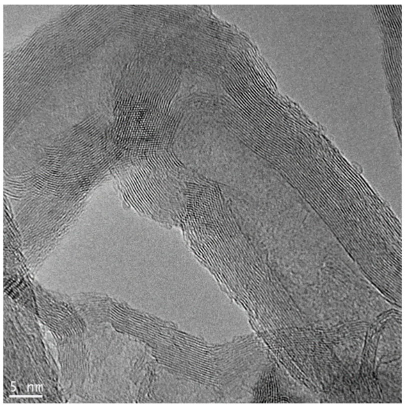

(e)

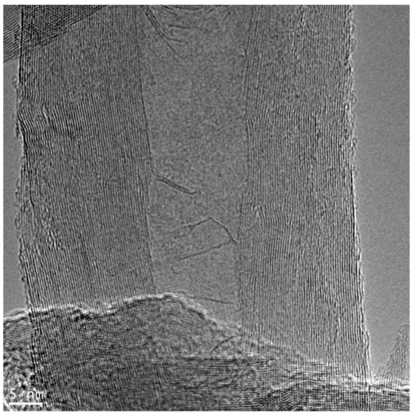

(f)

Figure S1. TEM images of treated samples of MWNTs: (**a**) MWNT-1020_1M; (**c**) MWNT-1020_3M; (**e**) MWNT-1020_6M; (**b**) MWNT-4060_1M; (**d**) MWNT-4060_3M; (**f**) MWNT-4060_6M.

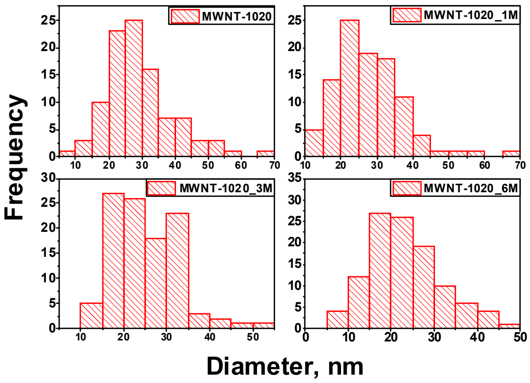

(a)

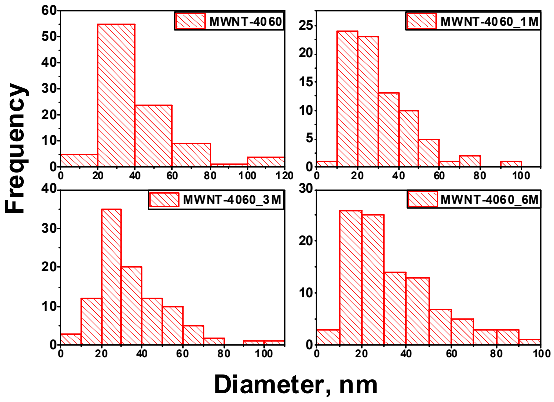

(b)

Figure S2. Distribution of diameters of CNFs formed during the treatment: (**a**) MWNT-1020 samples; (**b**) MWNT-4060 samples.

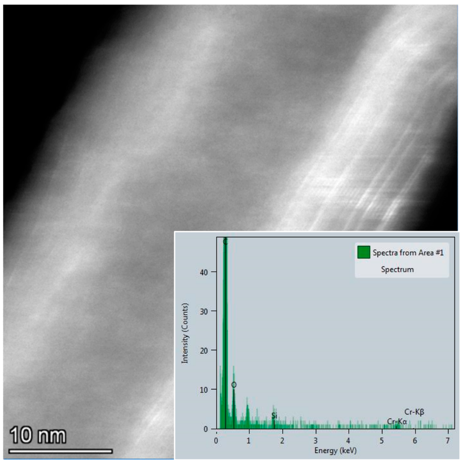

(a)

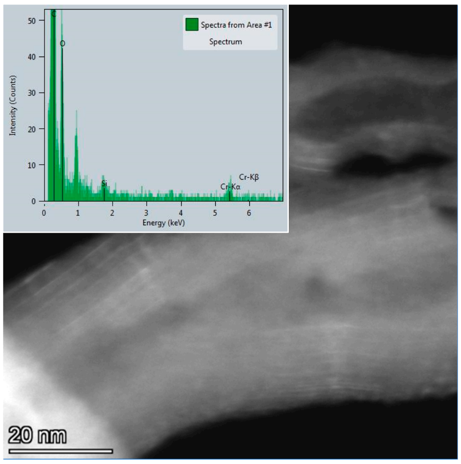

(b)

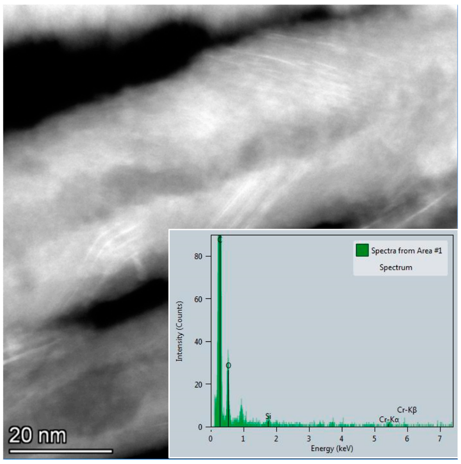

(c)

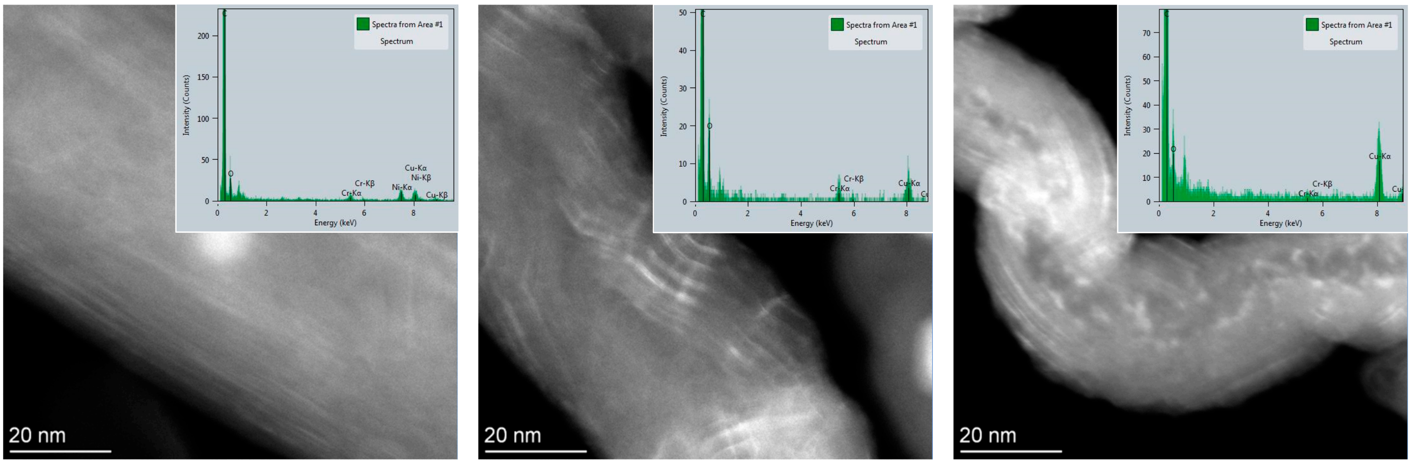

(d) (e) (f)

Figure S3. (**a-f**) HAADF-STEM micrographs of MWNT-4060_3M sample accompanied with EDX spectra.

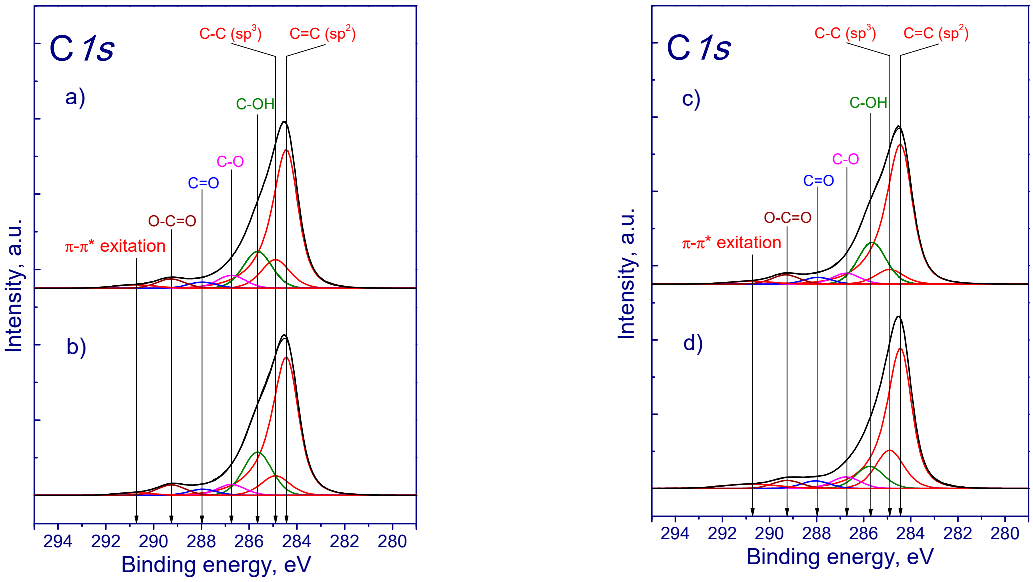

Figure S4. C1s X-ray photoelectron spectra of treated samples: (**a**) MWNT-4060_1M; (**b**) MWNT-1020_1M; (**c**) MWNT-4060_3M; (**d**) MWNT-1020_3M.

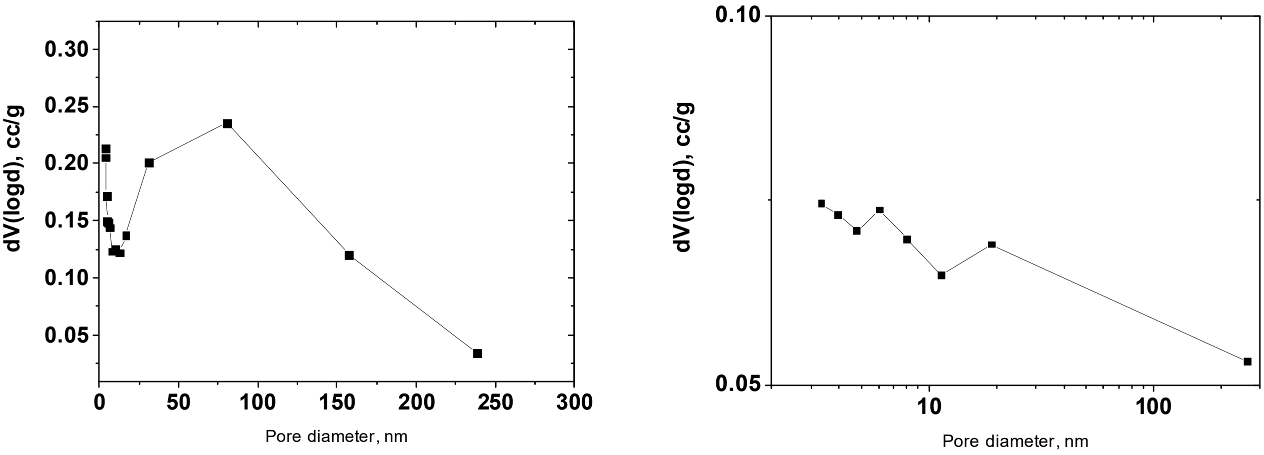

(**a**) (**b**)

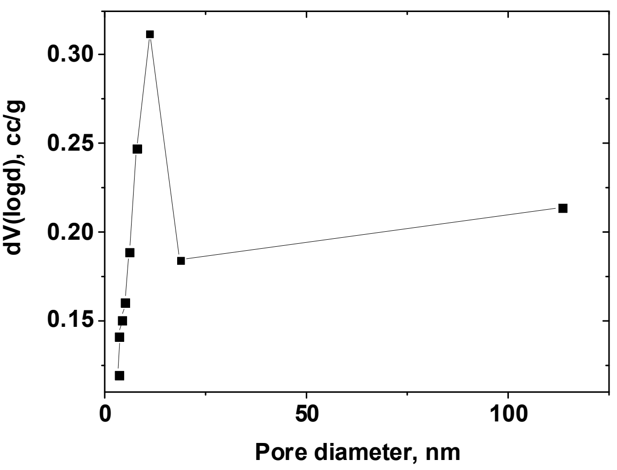

(c)

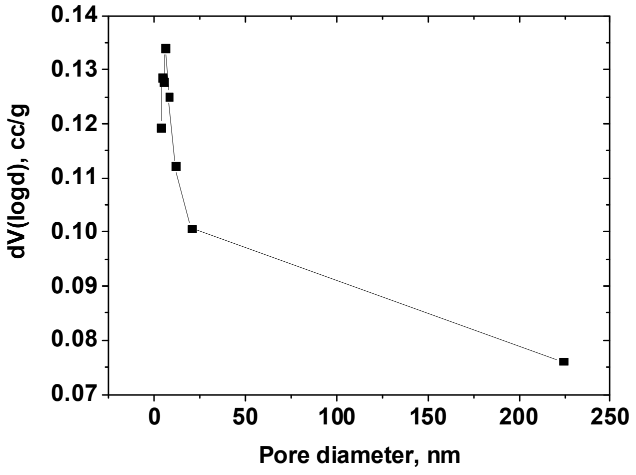

(d)

Figure S5. Pore size distribution of MWCNT samples with high specific capacitance: (a) WNT-1020; (**d**) MWNT-4060 initial samples; (**c**) MWNT-1020_6M; (**d**) MWNT-4060_3M chemically treated samples.
